# High Areal Capacity Porous Sn-Au Alloys with Long Cycle Life for Li-ion Microbatteries

**DOI:** 10.1038/s41598-020-67309-7

**Published:** 2020-06-26

**Authors:** Sai Gourang Patnaik, Ankita Jadon, Chau Cam Hoang Tran, Alain Estève, Daniel Guay, David Pech

**Affiliations:** 1 LAAS-CNRS, Université de Toulouse, CNRS, Toulouse, France; 2 INRS-Énergie, Matériaux et Télécommunications, Varennes, Québec, Canada

**Keywords:** Electronic devices, Batteries

## Abstract

Long-term stability is one of the most desired functionalities of energy storage microdevices for wearable electronics, wireless sensor networks and the upcoming Internet of Things. Although Li-ion microbatteries have become the dominant energy-storage technology for on-chip electronics, the extension of lifetime of these components remains a fundamental hurdle to overcome. Here, we develop an ultra-stable porous anode based on SnAu alloys able to withstand a high specific capacity exceeding 100 µAh cm^−2^ at 3 C rate for more than 6000 cycles of charge/discharge. Also, this new anode material exhibits low potential (0.2 V versus lithium) and one of the highest specific capacity ever reported at low C-rates (7.3 mAh cm^−2^ at 0.1 C). We show that the outstanding cyclability is the result of a combination of many factors, including limited volume expansion, as supported by density functional theory calculations. This finding opens new opportunities in design of long-lasting integrated energy storage for self-powered microsystems.

## Introduction

With ultrahigh speed rate and low latency of 5 G mobile networks in the upcoming years, the emergence of the Internet of Things (IoT) is set to revolutionize all aspects of our lives^1,2^. This trendy concept describes a network of connected objects able to collect data, interact with the environment and communicate wirelessly over the internet for a plethora of applications such as healthcare, self-driving vehicles, environmental monitoring and smart manufacturing. The integration of self-powered micrometric sensors will rely on efficient microscale energy storage units^3^ to interface with various types of energy harvesters, which are intermittent by nature. The inherent requirement is to enable monitoring by a remote sensor without further maintenance. Microbatteries with high areal capacity and ultralong life cycle are thus quintessential in such a scenario for realizing “fit and forget” type solutions. Moreover, microbatteries will be confined in an embedded microsystem with limited space available. The size and the compactness being critical, it is imperative to consider their properties normalized to the surface area. This calls for utilization of innovative materials with high volumetric capacity coupled with exploitive architectures, which are easy to realize and compatible with microsystem fabrication technologies, providing robust stability in limited footprint area. Furthermore, pricey electrode materials can be used in miniaturized devices, where cost is mainly determined by the microfabrication process and not by the minute amount of active materials involved.

Alloy anodes are promising candidates as negative electrodes in Li and post Li-ion chemistries due to their high specific capacity^4–9^. Especially, Sn and their oxides have extremely high volumetric capacity (7200 mAh cm^−3^ for Sn, *i.e*. even higher than metallic Li) and hence apt for utilization in microbatteries requiring high energy density per footprint area. However, Sn anodes suffer from a variety of issues like volume expansion (up to 360%), related continuous solid electrolyte interface (SEI) formation and capacity fading^10,11^. Such issues are magnified in context of microbatteries, where there are not many amenities for structural engineering due to limited space. Nevertheless, there have been several efforts to utilize Sn-based materials in microbatteries – through alloying with other metal^12–14^ constructing porous 3D architectures^15–21^ making homogenous/ordered carbon composites^22–26^ etc. However, most of them failed to achieve long cyclability and high rate capability. In the cases where high rate was demonstrated, the charge-discharge profile was more capacitive than battery-like^24^.

Here, we report on highly porous anodes based on Li_*x*_SnAu alloys with exceptional cycling stability (exceeding 6 000 cycles) and rate capability (>150 µAh/cm^2^ at 4 C) with flat discharge profile even at high rates. The tin gold alloy is homogeneously formed onto nanoporous structures prepared using the dynamic hydrogen bubble template (DHBT) method, by which 3D self-supported metallic structures, with a wide range of interconnected pores, are electrodeposited and sculptured by H_2_ bubbles generated at high overpotentials^27^. The obtained porous alloy film (10–100 μm thick) is conductive, evolves into further nanoporous structure upon reaction with Li and exhibits remarkable mechanical stability upon lithiation, even using a standard liquid-based electrolyte, with limited volume expansion as established by DFT modelling. This finding represents a major step forward towards the integration of high-energy long-cycling microbatteries for IoT applications.

### Porous SnAu electrodes

Highly porous metallic current collectors were first prepared using the dynamic hydrogen bubble template (DHBT) method on a silicon substrate. A key advantage of this technique is its cleanliness, simplicity, and ease of preparation, making the DHBT method easily transferable to pilot production line in microelectronic facilities. DHBT was used to prepare highly porous gold architectures onto which Sn was subsequently deposited (Supplementary Fig. 1). We have previously reported the successful formation of such highly porous gold films by constant potential mode^28,29^. However, for commercial upscaling, galvanostatic deposition is preferred and hence, in the present work, an optimized protocol with constant current deposition was utilized. Various parameters like concentration of Au^3+^ ions, acid concentration, deposition time and stability of the porous films over time were varied individually to reach a set of optimized deposition conditions (see Supplementary Fig. 2 for details). The thickness, *t*, apparent porosity, *p*, and aspect ratio, *AR*, (defined as the ratio between the electrochemical active surface area and the geometrical surface area) of porous gold films used in this study are *t* = 59 μm, *p* = 88.0% and *AR* = 900 cm^2^/cm^2^. This huge area to volume ratio allows more active material to be loaded per unit area of electrode and results in better interfacial kinetics and lower ohmic losses due to short transport distances^30–32^. Moreover, with such high porosity, little amount of gold is involved and cost of current collector material is estimated to be less than 30 cents cm^−2^. The porous nature of the resulting electrodeposited film can be observed in the scanning electron microscope (SEM) images of Supplementary Fig. 3, where macropores are visible within an interconnected network of pore walls. At higher magnification, an ensemble of numerous Au dendrites and nodules was observed. They are oriented in all directions, forming mechanically stable and self-supported pore walls.

**Figure 1:**
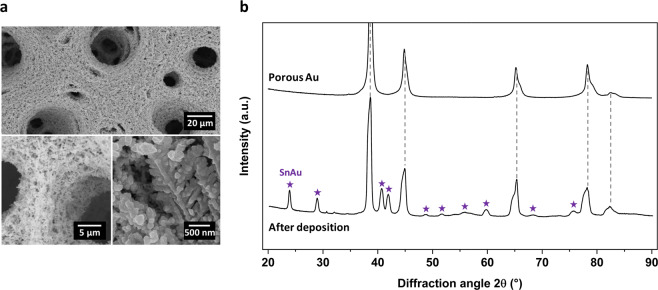
Characterization of the porous electrode. (**a)** SEM image after Sn electrodeposition with preservation of the highly porous structure. (**b)** Grazing incidence XRD pattern of the porous Au before and after Sn electrodeposition with several peaks matching SnAu alloy.

Following this, electrodeposition of Sn was achieved by constant potential deposition from acidic medium (pH <2.0) in order to realize porous Sn based electrodes having flat charge-discharge plateaus due to formation of self-nanoporous structure upon lithiation^33^. From SEM images (Fig. 1a), the morphology of the porous electrode was not affected by the deposition of tin. Its honeycomb-like structure is preserved with similar pore sizes and dendritic network as before Sn electrodeposition. From the color distribution of energy-dispersive X-ray (EDX) imaging (Supplementary Fig. 4), both Sn and Au elements are observed at every location, suggesting a homogeneous and conformal deposition of Sn on Au, including dendrites and nodules. Grazing incidence X-ray diffraction (GI-XRD) measurements were also performed before and after Sn electrodeposition (Fig. 1b). Surprisingly, the formation of SnAu alloy occurred under ambient conditions during the electrodeposition process, with no XRD peaks related to Sn detected. Such phenomenon has recently been observed elsewhere^34^ and is mostly related to the nature of porous gold, which is different from bulk gold or thin films of gold. The spontaneous formation of SnAu alloy can be explained by the fact that under sufficient reductive potentials interaction between both surface adsorbed Sn and dissolved Sn with the gold substrate exceeds the binding energy between the Sn atoms. As a result, Sn deposition on gold leads to place exchange and hence results in surface alloying^35,36^. Similar electrodeposition process carried out on Au thin films prepared by physical vapor deposition (PVD) resulted in pure Sn phases rather than SnAu alloys (Supplementary Fig. 5). This is because for non-porous gold thin films, a limited amount of Au atoms is available for SnAu alloy formation by interaction with Sn^2+^ ions. Once the gold atoms at the surface of a non-porous Au thin films have reacted with Sn resulting in SnAu alloy formation, further deposition results in the formation of pure Sn as no more pure Au atoms are available for formation of SnAu alloy. However, during slow electrodeposition of Sn on porous gold from dilute solutions, the amount of Au is very large, thus giving rise to the formation of only the SnAu phase with no metallic Sn. Therefore, both from EDX and XRD analysis, we conclude to a conformal formation of SnAu alloy onto a porous framework.

### Characterization of the different phase transitions

Upon preliminary electrochemical investigation, the achieved reversible capacity for Sn on porous Au scaffolds was much higher than the theoretical capacity of Sn anodes (~990 mAh g_(Sn)_^−1^) by considering deposited weight of Sn alone as active material (Supplementary Fig. 6a). This was puzzling because, even though Au lithiates to some extent, it does not provide high reversible capacity with Li ions^37^. Hence, as stated before from XRD studies, porous SnAu alloy has to be considered as the active material rather than only Sn. SnAu based alloys have been well studied in the past as solder materials in the semiconductor industry due to their thermal stability, mechanical properties and creep resistance^38–40^. In the δ phase, SnAu is an equiatomic intermetallic compound having a hexagonal crystal structure with high elastic modulus and superior fatigue resistance. Hence, considering the active material to be SnAu alloy, the specific gravimetric capacity is around 650 mAh g_(SnAu)_^−1^ (Supplementary Fig. 6b) which explains the anomaly of higher than theoretical capacity of Sn anodes. It is worth noting that such high gravimetric value is associated with very good coulombic efficiency even at very high C-rates.

**Figure 2:**
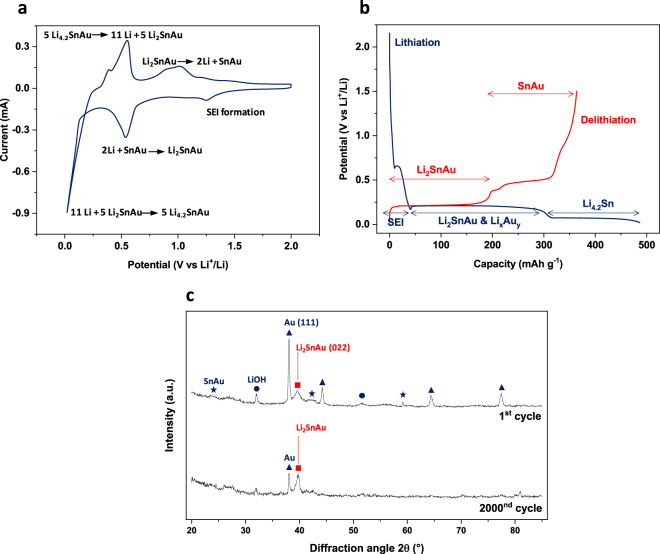
Phase transitions upon lithiation of SnAu porous alloy. (**a)** Cyclic voltammogram(1^st^ cycle) in half-cell set-up using LiPF_6_ in EC:DEC electrolyte at 0.1 mV s^−1^. (**b)** Galvanostatic lithium insertion/extraction profiles (1^st^ cycle) at C/10 with two flat plateaus. (**c)** *Ex situ* XRD pattern recorded after 1 cycle and 2000 cycles of charge/discharge. After a certain number of cycles, only a stable Li_2_SnAu phase remains for lithiation.

To understand the mechanism of lithiation of SnAu alloy, cyclic voltammetry studies were performed at low scan rate (Fig. 2a and Supplementary Fig. 7). The electrodes exhibit a two-step lithiation/delithiation process akin to other Sn based Gr-11 intermetallic alloys like SnCu^17^. The overall reaction can be written as follows, with associated calculated capacity:

1 $${\rm{SnAu}}+2{\rm{Li}}\leftrightarrow {{\rm{Li}}}_{2}{\rm{SnAu}}( \sim \,170\,{{\rm{mAhg}}}^{-1})$$

2 $${{\rm{Li}}}_{2}{\rm{SnAu}}+x\,{\rm{Li}}\leftrightarrow {{\rm{Li}}}_{2+x}{\rm{SnAu}}( \sim \,179\,{{\rm{mAhg}}}^{-1}\,{\rm{for}}\,x=2.2)$$

This is also in agreement with galvanostatic charge discharge measurements (Fig. 2b and Supplementary Fig. 8). The lithiation of SnAu alloy anodes occurs through the formation of the SEI, followed by reaction (1) and finally alloying with Li, reaction (2), which contributes to maximum of total charge capacity. The first plateau at 0.2 V vs. Li^+^/Li (Fig. 2b) also includes some amount of lithiation of unalloyed gold, thereby exceeding the capacity from reaction (1). The contribution of capacity from individual reactions in the charge/discharge profile fits in with theoretically estimated capacities, providing reaffirmation of the reaction mechanism. This was also in agreement with the findings from XRD studies of the cycled electrodes (Fig. 2c). The electrode after one cycle of lithiation-delithiation is constituted of SnAu, its lithiated counterpart *i.e*. Li_2_SnAu, and unalloyed Au underneath, as indicated by the XRD spectrum. After a large number of 2 000 cycles, the peaks corresponding to Li_2_SnAu and unalloyed Au are quite conspicuous, whereas SnAu peak has completely disappeared. This indicates that even during long cycling, the porous conducting 3D scaffold of gold stays intact, providing the much-needed electronic conductivity. Also, as the SnAu signal almost completely disappeared, it indicates that only reaction (2) is now providing the reversible capacity. In addition, it is known that the stannides of the family Li_*x*_Sn_*y*_Au_*z*_ (for e.g. Li_2_Sn_2_Au has D_Li(max)_ = 1.5 × 10^−6^ cm^2^ s^−1^ at 25 °C) exhibit good Li^+^ diffusion coefficients even at low Li content^41^ and hence, the presence of Li_2_SnAu after long cycling might also possess efficient Li^+^ transport properties in the electrode. The electrochemical lithiation of conformally deposited SnAu alloy not only gives high reversible specific areal capacity, but also results in intermediates that favor Li^+^ ion diffusion kinetics, displaying its superiority as a prospective anode material for Li-ion microbatteries.

### Electrochemical performances

**Figure 3:**
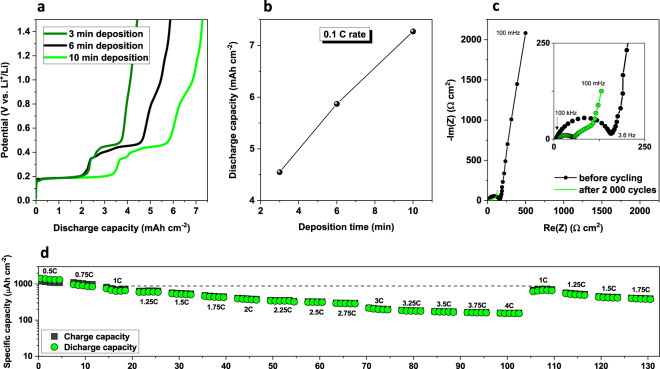
Areal electrochemical performances. (**a,b)** Discharge areal capacity at low C-rate (C/10) of SnAu alloys synthesized with increasing electrodeposition time. Huge areal capacities ranging from 4.6 to 7.3 mAh cm^−2^ are obtained. (**c)** Corresponding Nyquist plot before and after 2 000 cycles. (**d)** C-rate performance from C/2 to 4 C for a 10 min electrode.

The specific capacity per unit area and the long-term stability of the anode have been investigated using a conventional LiPF_6_ based liquid electrolyte without any additives for demonstration effects (Fig. 3). Figure 3a,b show the comparison of first discharge curves of porous SnAu electrodes for different deposition times. The electrodes exhibit extraordinarily high specific capacity at low C-rate, ranging from 4.6 to 7.3 mAh cm^−2^ during first discharge, which is much higher than most reported microbattery anodes (see Supplementary Table 1), highlighting the high surface area for charge storage. As expected, the capacity increases linearly from 3 to 10 min of electrodeposition due to the increase of the deposited active material.

Electrochemical impedance spectroscopy (EIS) studies were performed before cycling and after 2 000 cycles (Fig. 3c). The overall impedance is reduced after cycling, indicating enhanced charge transfer kinetics after cycling. This is in agreement with formation of Li_2_SnAu (which has good Li ion diffusion kinetics) and consistent with a homogeneous distribution of the active material within unalloyed porous Au framework which provides good electronic conductivity to the matrix even after long cycling. C-rates studies were also carried out to see the robustness of the electrode towards rate fluctuations (Fig. 3d). The electrode shows extraordinary stability towards rate change with 158 μAh cm^−2^ at 4 C rate and reversible capacity retention when subsequently cycled at lower rates (816 μAh cm^−2^ at 1 C).

**Figure 4:**
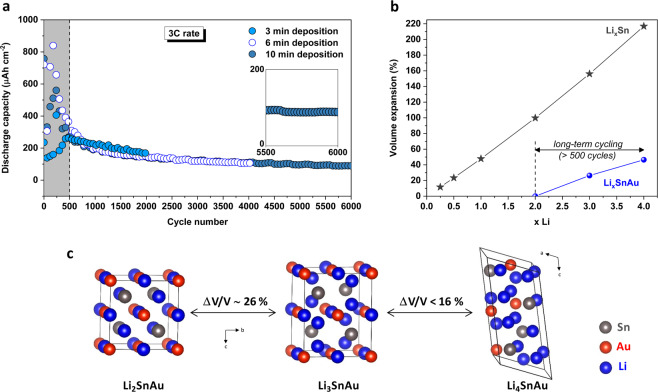
Investigation of the volume expansion of Li_*x*_SnAu compounds. (**a)** Cycling performances of different Li_*x*_SnAu electrodes (including pre-cycling treatment in shading area) at 3 C up to 2000, 4000 and 6000 cycles. Very good stability is obtained after ∼500 cycles of charge-discharge. (**b)** Comparison of the volume expansion of Li_*x*_SnAu with Li_*x*_Sn based on DFT calculations. (**c**) Representative structures of Li_2_SnAu, Li_3_SnAu and Li_4_SnAu associated with volume change.

Extra long-term cyclability of the electrodes was evaluated at 3 C rate due to time constraints, using samples synthesized with different electrodeposition times (Fig. 4a). The electrodes exhibit excellent stability testifying the reversible transition from Li_2_SnAu to Li_4.2_SnAu. More impressively, the samples feature superior cyclability, sustaining more than 6000 cycles with an average capacity decay below 10 nAh cm^−2^ per cycle. During initial cycles, decrease in specific capacity can be ascribed to rearrangements in the electrode due to volume expansion, which exposes new surface for lithiation and continuous SEI formation. However, after the pre-cycling treatment, the discharge capacity was very stable due to homogeneous distribution of the lihtiated intermediates within the electron conducting porous gold framework and limited volume expansion, which is further corroborated by DFT studies.

### Density functional theory (DFT) studies

To further elucidate the origin of the phenomenal stability of lithiated tin gold electrode, we have compared the volume expansion/contraction of Li_*x*_Sn and Li_*x*_SnAu during the lithiation/delithiation process using DFT calculations (Fig. 4b). In the case of Sn, it evolves through different phases upon lithiation from pure Sn to Li_17_Sn_4_ ^42^, meaning that the number of atoms of lithium *x* accommodated into each Sn atom varies from 0 to 4.25 during each cycling. Such continuous increase/decrease in Li-concentration leads to dramatical volume expansion of Li_*x*_Sn (with respect to the volume of Sn, Fig. 4b)^43^, causing mechanical degradation such as cracking and pulverization.

Regarding SnAu alloy, as stated before, its first lithiations lead to the formation of Li_2_SnAu compounds. This is consistent with the behaviour observed in the shading areas of Fig. 4a showing the pre-cycling treatments of different Li_*x*_SnAu electrodes. All samples behave similarly: huge discharge capacities accompanied with strong irregularities are first observed with decay in reversible capacity for a regime going from 0 to ~500 cycles. This initial erratic trend in discharge capacity can be partly ascribed to rearrangements in the electrode at the nano regimes during cycling, thereby exposing new surfaces for lithiation, as well as a progressive quasi-irreversible transformation of SnAu into Li_2_SnAu (reaction (1)). DFT shows that this reaction involves different phase transformations (orthorhombic, hexagonal and cubic). This “pre-treatment” regime is then quickly followed by an extreme stability for the rest of the lifetime of the cells, whatever the mass loadings of the electrode (Fig. 4a). Taking into account reaction (2), long-term cycling involves afterwards exclusively the lithiation of Li_2_SnAu into further Li_*x*_SnAu lithiated phases, with an *x* value varying between 2 to ∼4. Figure 4c shows the structural evolution of Li_2_SnAu upon further lithiation along with associated volume change. Unlike Li_*x*_Sn, the variation in volume during the lithiation/delithiation process has to be relative to the volume of the starting Li_2_SnAu material. The first lithiation of Li_2_AuSn has minor volume change of ~26% with following steps having lower associated volume expansion while transitioning from individual lithiated phases. The transition from Li_2_SnAu to Li_4_SnAu (occurring during very long cycling) is therefore associated with a volume change which is more than 5 times lower than the one related to the conversion of Sn to Li_4_Sn. Moreover, the void space of the porous structure can accommodate this limited volume expansion of the active material, making porous Li_2_SnAu a very promising anode for long-term cycling microbatteries.

## Conclusions

In the current work, we have reported 3D porous current collector based microbattery architecture for ultra-long life cycle (>6000 cycles) and extremely high areal capacity (>7 mAh cm^−2^) anodes. The entire 3D structure is compact (<0.8 cm^2^) and can be assembled on silicon wafers to facilitate their microelectronics based integration. Conformal coating of these highly porous substrates with active materials (SnAu) is also realized under ambient conditions through simple electroplating technique to ensure ease of further upscaling. Explanations over origin of high stability and mechanism of charge storage are also provided from materials/electrochemical characterizations and DFT simulations, thus opening a niche for further development of these 3D porous engineered architectures. Further research focus on upgradation of these 3D micro-architectures will be to fabricate full devices in interdigitated configuration with solid electrolytes, develop efficient packaging routes and trail integration with other microelectronic components. Moreover, replacing the liquid electrolyte with a solid electrolyte should further enhance the stability and durability, giving rise to even higher energy storage and cyclability. The work also illustrates the utilization of significantly different material chemistry for microbatteries, which can be investigated further by changing individual elements and analyzing their electrochemical kinetics. Overall, we demonstrate successful microengineering of 3D porous architectures for Li-ion microbatteries with tremendous potential as integrative miniature power sources for microdevices.

## Methods

### Material synthesis

A Ti(100 nm)/Au(300 nm) thin film was deposited by evaporation on an oxidized silicon substrate and electrochemically pretreated by cycling the potential at a scan rate of 100 mV s^−1^ between −0.3 and +1.7 V versus saturated calomel electrode (SCE) in 1 M H_2_SO_4_ until a stable voltammogram was obtained. Porous metallic current collectors were prepared using the DHBT technique from an optimized solution of 2 × 10^−3^ M of HAuCl_4_.3H_2_O in 3 M H_2_SO_4_ by applying 5 A cm^−2^ for 20 min in a 3-electrode configuration. The porous Au film was then washed several times in de-ionized water before vacuum drying for 30 min. SnAu alloy formation was then carried out by electrodeposition from a freshly prepared solution of 7.5 × 10^−3^ M of SnCl_2_ in 0.02 M HCl by applying -2 V *vs*. SCE for 1, 3, 6, 10 min with mass loadings of ∼2.9 mg cm^−2^ of SnAu per min. The electrodes (0.8 cm^2^) were further dried to remove any moisture content and tested using Li-ion half-cells (EL-Cell) assembled in a glove box with purified argon, with lithium foil as counter and reference electrodes, and glass fiber separator soaked with 1 M LiPF_6_ in ethylene carbonate (EC)/diethyl carbonate (DEC) (1:1 volume ratio).

### Characterizations

The electrochemical synthesis and characterizations were performed with a VMP-3 and a VSP Biologic potentiostat connected to an external 10 A booster channel. The amount of Sn reduced was calculated using Faraday’s law. Subsequently, assuming all of the reduced Sn underwent alloying with Au to form SnAu (since there was no peak corresponding to pure Sn in XRD for any of the samples), the weight of active material was obtained. The content ratio of SnAu (1:1) was obtained from XRD measurements. This was also confirmed from the obtained capacity which matched the theoretical capacity for lithiation of SnAu. For long life cycle studies, the cells were initially pre-cycled in the same potential range (0.02–1.5 V *vs*. Li/Li^+^) for two cycles at 0.1 C (0.1 mA cm^−2^) before ramping the rate. The surface morphology of the electrodes was examined by scanning electron microscopy (SEM) on a Hitachi S-4800 field emission electron microscope. The crystallographic structures were analyzed by grazing incidence X-ray diffraction (XRD) measurements on a Bruker D8 Advanced X-ray diffractometer with Cu K_α_ radiation (1.54184 Å), operating at 40 kV and 40 mA. DFT-based Vienna ab initio simulation package (VASP) was employed to perform calculations, using Perdew, Burke, and Ernzerhof (PBE) method for treating exchange and correlation. The valence electrons were described with a planewave basis set. Nuclei and core electrons were treated with pseudo-potentials of the projector augmented wave type. The aspect ratio *AR* of the metallic current collector was defined as follows:

$$AR=\frac{Electrochemical\,Active\,Surface\,Area\,(EASA)}{Geometrical\,Area}$$

The electrochemical surface area of porous gold was calculated using the charge associated with the reduction of gold oxide, using a value of 390 μC cm^−2^ ^44^ The porosity *p* (%), describing the fraction of void space in the material, was calculated using the following equation:

$$p( \% )=\frac{{V}_{p}}{V}=\frac{(S\times t)-(\frac{w}{{\rho }_{Au}})}{(S\times t)}$$

where *V*_*p*_ is the pore volume, *V* the apparent volume of the electrode, *S* the geometric electrode surface, *t* the film thickness, *w* the weight of the deposited gold and *ρ*_*Au*_ the density of gold set at 8.9 g cm^−3^.

## Supplementary information

Supplementary information.

## Data Availability

The data that support the plots within this paper can be obtained free of charge from Zenodo via https://zenodo.org.
