# Supplementary material for: High Areal Capacity Porous Sn-Au Alloys with Long Cycle Life for Li-ion Microbatteries
Source: Sci Rep. 2020 Jun 26;10:10405. doi: 10.1038/s41598-020-67309-7 (PMC7320134; doi:10.1038/s41598-020-67309-7)
Supplement: Supplementary file 1 — Supplementary information. [file 41598_2020_67309_MOESM1_ESM.docx]

**SUPPLEMENTARY MATERIALS**

**High Areal Capacity Porous Sn-Au Alloys with Long Cycle Life for Li-ion Microbatteries**

Sai Gourang Patnaik, Ankita Jadon, Chau Cam Hoang Tran, Alain Estève, Daniel Guay & David Pech^*^

*E-mail : [dpech@laas.fr](mailto:dpech@laas.fr)


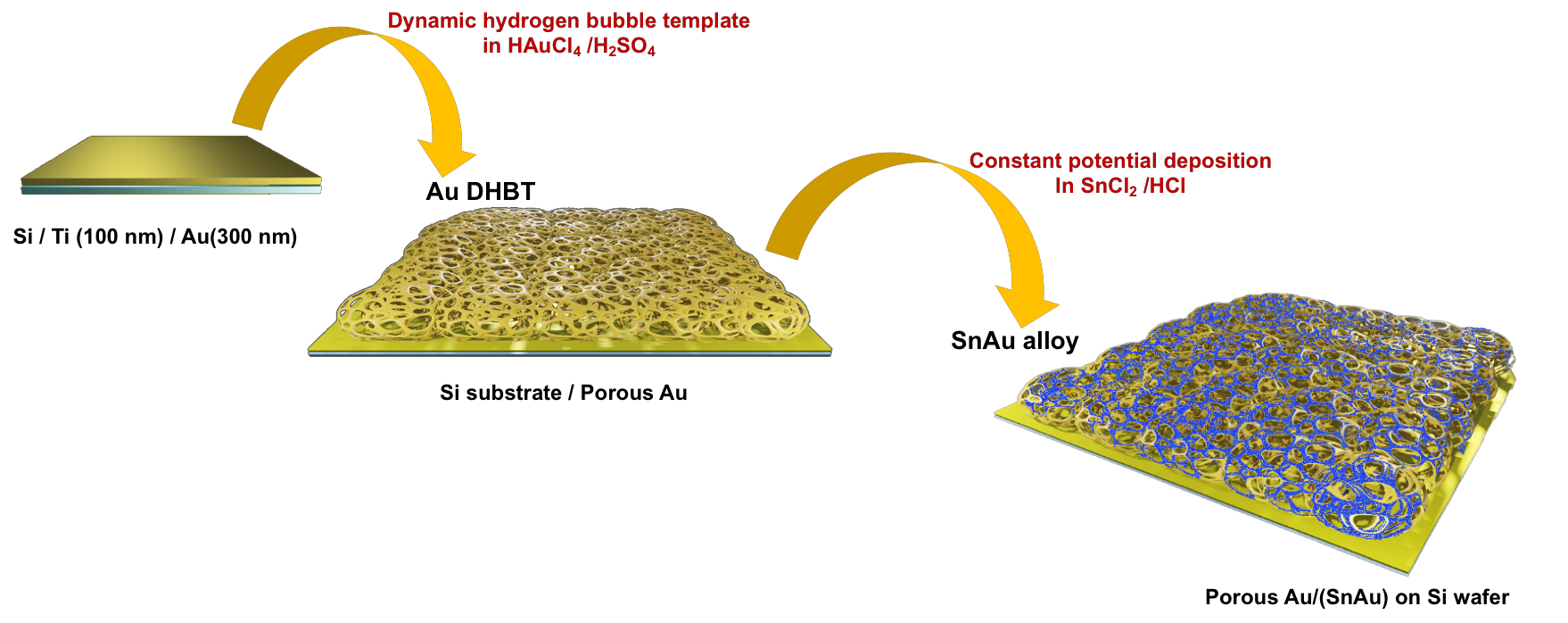


**Supplementary Fig. 1 ǀ Synthetic diagram for porous SnAu alloy formation through electrodeposition on porous gold substrate.**

**Supplementary Fig. 2 ǀ** **Porous Au synthesis using different DHBT parameters.**
**a, b,** Thickness, aspect ratio and porosity of the deposited film with varying current density for 10 min deposition in 2mM of HAuCl_4_.3H_2_O / 3M H_2_SO_4_. **c, d,** Influence of the deposition time at 5 A cm^-2^.

**Supplementary Fig. 3 ǀ** **SEM image of a porous Au before Sn electrodeposition.** The images at different magnifications shows a highly porous structure with different levels of pores.

**Supplementary Fig. 4 ǀ** **EDX analysis of a porous Au after 10 min of Sn electrodeposition. a, b, c,** EDX mapping images of Au, O and Sn elements suggesting uniform deposition of Sn-based alloy on Au. **d,** EDX spectrum of the electrode before lithiation.

**Supplementary Fig. 5 ǀ GI-XRD pattern after Sn electrodeposition onto a flat PVD Au (300 nm).** The XRD peaks show only the presence of Sn metal with no SnAu signal.

**Supplementary Fig. 6 ǀ Gravimetric capacities of the electrode at different current densities. a,** Gravimetric performances normalized by the weight of Sn alone. **b,** Gravimetric performances normalized by the weight of SnAu. The electrode was tested after 1 min electrodeposition.


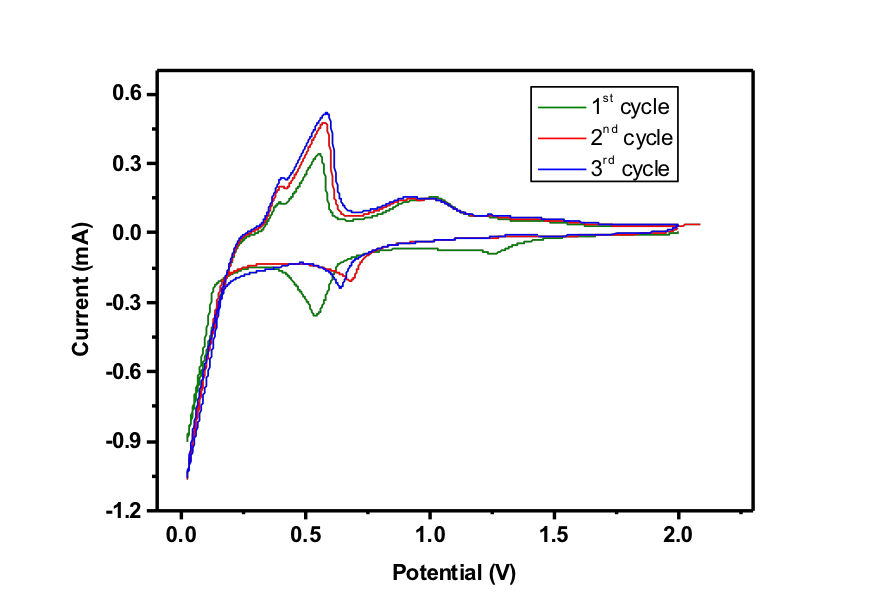


**Supplementary Fig. 7 ǀ Cyclic voltammogram (different cycles) in half-cell set-up using LiPF_6_ in EC:DEC electrolyte at 0.1 mV s^-1^ for a 1 min electrode.**


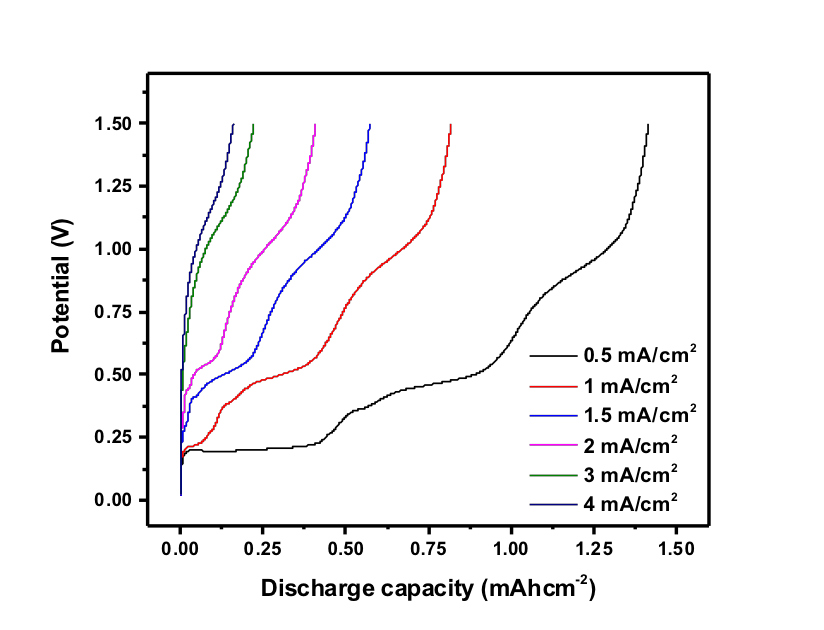


**Supplementary Fig. 8 ǀ Galvanostatic discharge profiles at different C rates for a 10 min electrode.**

| Electrode material | Areal capacity | Reported lifetime | Potential | Electrolyte | Reference |
| --- | --- | --- | --- | --- | --- |
| Carbon  (C-MEMS technology) | 125 μAh cm^-2^ | - | 0.3 V *vs.* Li^+^/Li | - | S1 |
| Polycrystalline Si  (3D Si microcontainers) | 255 μAh cm^-2^ (C/10) | - | 0.2 V *vs.* Li^+^/Li | 1M LiPF_6_ in EC/DEC/DMC | S2 |
| NbO_2_ nanoparticles  on C foam | 0.7 mAh cm^-2^ | 20 cycles | 1–3 V *vs.* Li^+^/Li | 1M LiPF_6_ in EC/DEC | S3 |
| TiO_2_ on Al nanorods | 11 μAh cm^-2^ (C/5) | 50 cycles | 1.8 V *vs.* Li^+^/Li | 1M LiClO_4_ in PC | S4 |
| Ni/TiO_2_ nanowire | 120 μAh cm^-2^ (C/3) | 50 cycles | 1.8 V *vs.* Li^+^/Li | 1M LiPF_6_ in EC/DMC | S5 |
| Ni-Sn nanowires | 4.6 mAh cm^-2^ (C/5) | 50 cycles | 0.01–1.5 V *vs.* Li^+^/Li | 1M LiPF_6_ in EC/DMC | S6 |
| Cu_2_Sb nanopillars | 125 μAh cm^-2^ (C/50) | 50 cycles | 0.8 V *vs.* Li^+^/Li | Gel PEA/PPGDA/LiTFSI | S7 |
| Ni core / NiO shell | 0.5 mAh cm^-2^ (1C) | 50 cycles | 0.01–3 V *vs.* Li^+^/Li | 1M LiPF_6_ in EC/DEC | S8 |
| Stacked Ge/Cu nanowire | 16 mAh cm^-2^ (C/10) | 50 cycles | 0.01–1.5 V *vs.* Li^+^/Li | 1M LiPF_6_ in EC/DEC | S9 |
| Fe_2_O_3_ on N-doped carbon nanotubes | ~1.2 mAh cm^-2^ | 60 cycles | 0.05–2 V *vs.* Li^+^/Li | 1M LiPF_6_ in EC/DEC | S10 |
| Anodized Ti_3_SiC_2_ | 380 μAh cm^-2^ | 60 cycles | 2–3 V *vs.* Li^+^/Li | 1M LiPF_6_ in EC/DEC | S11 |
| PANI coated Au nanotubes | 32 μAh cm^-2^ | 75 cycles | 2–3.6 V *vs.* Li^+^/Li | 1M LiClO_4_ in EC/DEC | S12 |
| SnO_2_ on Ni nanofoams | 0.5 mAh cm^-2^ | 100 cycles | 0.002–0.9 V *vs.* Li^+^/Li | 1M LiPF_6_ in EC/DEC | S13 |
| Multi-layered Si/C films | 300 μAh cm^-2^ | 100 cycles | 0.02–1.5 V *vs.* Li^+^/Li | 1M LiPF_6_ in EC/DEC | S14 |
| Nb alloyed TiO_2_ nanotubes | 50 μAh cm^-2^ | 100 cycles | 1–3 V *vs.* Li^+^/Li | 1M LiPF_6_ in EC/DEC | S15 |
| MoS_2_ on TiO_2_ nanotube | 440 μAh cm^-2^ (1C) | 100 cycles | 0.01–3 V *vs.* Li^+^/Li | 1M LiPF_6_ in EC/DEC | S16 |
| 3D Ni phosphide foam | 356 μAh cm^-2^ | 100 cycles | 1–3 V *vs.* Li^+^/Li | 1M LiPF_6_ in EC/DEC | S17 |
| Nanoarchitectured Ge | 1.5 mAh cm^-2^ | 140 cycles | - | 1M LiPF_6_ in EC/DEC/PC | S18 |
| NiSn on porous Ni | 1.2 mAh cm^-2^ (C/20) | 200 cycles | 0.02–1.5 V *vs.* Li^+^/Li | 1M LiClO_4_ in EC/DEC | S19 |
| TiO_2_ on Si microtubes | 200 μAh cm^-2^ (C/10) | 230 cycles | 1-2.5 V *vs.* Li^+^/Li | 1M LiTFSI in EC/DEC | S20 |
| TiO_2_ nanotube array | 22 μAh cm^-2^ | 6000 cycles | 1-2.6 V *vs.* Li^+^/Li | 1M LiPF_6_ in EC/DEC/DMC | S21 |
| Porous Li_2_SnAu | **7.3 mAh cm^-2^ (C/10)** | **> 6000 cycles** | **< 0.1 V *vs.* Li^+^/Li** | **1M LiPF_6_ in EC/DMC** | **This work** |

*EC: ethylene carbonate, DEC: diethyl carbonate, DMC: dimethyl carbonate, PC: propylene carbonate, PEA: polyetheramine, PPGDA: polypropylene glycol diacrylate.*

**Supplementary Table 1 ǀ Overview of the performances of negative electrodes (half-cells) reported within the literature for Li-ion microbatteries.**

**Supplementary references**

S1. Wang, C. *et al.* C-MEMS for the manufacture of 3D microbatteries. *Electrochem. Solid-State Lett.* **7,** A435-A438 (2004).

S2. Baggetto, L., Knoops, H. C. M., Niessen, R. A. H., Kessels, W. M. M. & Notten, P. H. L. 3D negative electrode stacks for integrated all-solid-state lithium-ion microbatteries. *J. Mater. Chem.* **20,** 3703-3708 (2010).

S3. Asfaw, H. D., Tai, C.-W., Nyholm, L. & Edström, K. Over-stoichiometric NbO_2_ nanoparticles for a high energy and power density lithium microbattery. *ChemNanoMat* **3,** 646-655 (2017).

S4. Cheah, S. K. *et al.* Self-supported three-dimensional nanoelectrodes for microbattery applications. *Nano Lett.* **9,** 3230-3233 (2009).

S5. Wang, W. *et al.* Three-dimensional Ni/TiO_2_ nanowire network for high areal capacity lithium ion microbattery applications. *Nano Lett.* **12,** 655-660 (2011).

S6. Tian, M., Wang, W., Wei, Y. & Yang, R. Stable high areal capacity lithium-ion battery anodes based on three-dimensional Ni-Sn nanowire networks.*J. Power Sources* **211,** 46-51 (2012).

S7. Tan, S., Perre, E., Gustafsson, T. & Brandell, D. A solid state 3-D microbattery based on Cu_2_Sb nanopillar anodes. *Solid State Ion.* **225,** 510-512 (2012).

S8. Vlad, A. *et al.* Three-dimensional interconnected Ni_core_-NiO_shell_ nanowire networks for lithium microbattery architectures. *J. Mater. Chem. A* **4,** 1603-1607 (2016).

S9. Chang, W.-C., Lu, S.-P., Chu, H.-C. & Tuan, H.-Y. Lithium-ion battery anodes of stacked nanowire laminate for ultrahigh areal capacities. *ACS Sustainable Chem. Eng.* **7,** 156-164 (2019).

S10. Sharifi, T. *et al.* Hierachical self-assembled structures based on nitrogen-doped carbon nanotubes as advanced negative electrodes for Li-ion batteries and 3D microbatteries. *J. Power Sources* **279,** 581-592 (2015).

S11. Tesfaye, A. T. *et al.* Anodized Ti_3_SiC_2_ as an anode material for Li-ion microbatteries. *ACS Appl. Mater. Interfaces* **8,** 16670-16676 (2016).

S12. Gowda, S. R., Mohana Reddy, A. L., Zhan, X., Jafry, H. R. & Ajayan, P. M. 3D nanoporous nanowire current collectors for thin film microbatteries. *Nano Lett.* **12,**1198-1202 (2012).

S13. Haag, J. M., Pattanaik, G. & Durstock, M. F. Nanostructured 3D electrode architectures for high-rate Li-ion batteries. *Ad. Mater.* **25**, 3238-3243 (2013).

S14. Garino, N., Biserni, E., Li Bassi, A., Bruno, P. & Gerbaldi, C. Mesoporous Si and multi-layered Si/C films by pulsed laser deposition as Li-ion microbattery anodes. *J. Electrochem. Soc.* **162,** A1816-A1822 (2015).

S15. Salian, G. D. *et al.* Nobium alloying of self-organized TiO_2_ nanotubes as an anode for lithium-ion microbatteries. *Adv. Mater. Technol.* **3,** 1700274 (2018).

S16. Sopha, H. *et al.* ALD growth of MoS_2_ nanosheets on TiO_2_ nanotube supports. *FlatChem* **17,** 100130 (2019).

S17. Records, W. C., Wei, S. & Belcher, M. A. Virus-templated nickel phosphide nanofoams as additive-free, thin-film Li-ion microbattery anodes. *Small* **15,** 1903166 (2019).

S18. Lee, G.-H., Lee, S., Lee, C. W., Choi, C. & Kim, D.-W. Stable high-areal-capacity nanoarchitectured germanium anodes on three-dimensional current collectors for Li ion microbatteries. *J. Mater. Chem. A* **4,** 1060-1067 (2016).

S19. Zhang, H., Shi, T., Wetzel, D. J., Nuzzo, R. G. & Braun, P. V. 3D scaffolded nickel-tin Li-ion anodes with enhanced cyclability. *Adv. Mater.* **28,** 742-747 (2016).

S20. Eustache, E. *et al.* Silicon-microtube scaffold decorated with anatase TiO_2_ as a negative electrode for a 3D lithium-ion microbattery. *Adv. Energy Mater.* **4,** 1301612 (2014).

S21. Cai, C., Sun, F. & Xu, Y. Ultra-long life of TiO_2_ nanotube array microelectrode for Li-ion microbatteries. *Ionics* **24,** 2227-2232 (2018).
